# Supplementary material for: Garlic Consumption and All-Cause Mortality among Chinese Oldest-Old Individuals: A Population-Based Cohort Study
Source: Nutrients. 2019 Jun 30;11(7):1504. doi: 10.3390/nu11071504 (PMC6683033; doi:10.3390/nu11071504)
Supplement: Supplementary file 1 [file nutrients-11-01504-s001.pdf]

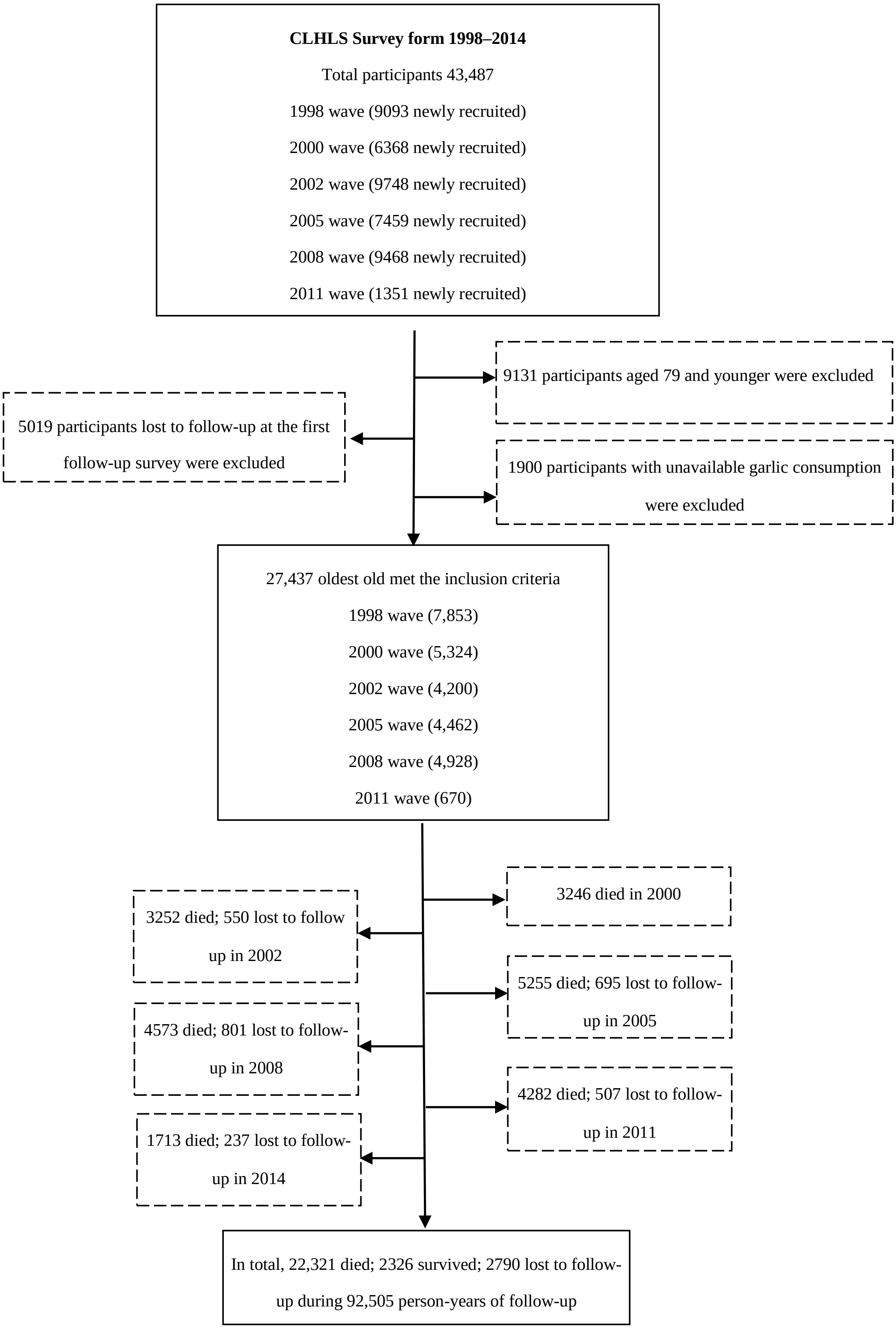

Figure S1. Cohort Selection Criteria, CLHLS Survey from 1998 to 2014. CLHLS: Chinese Longitudinal Healthy Longevity Study.
